# Mutations in *DNMT3A*, *U2AF1*, and *EZH2* identify intermediate-risk acute myeloid leukemia patients with poor outcome after CR1

**DOI:** 10.1038/s41408-017-0040-9

**Published:** 2018-01-10

**Authors:** Caner Saygin, Cassandra Hirsch, Bartlomiej Przychodzen, Mikkael A. Sekeres, Betty K. Hamilton, Matt Kalaycio, Hetty E. Carraway, Aaron T. Gerds, Sudipto Mukherjee, Aziz Nazha, Ronald Sobecks, Christopher Goebel, Donna Abounader, Jaroslaw P. Maciejewski, Anjali S. Advani

**Affiliations:** 1 Department of Hematology and Oncology, Taussig Cancer Institute, Cleveland Clinic, Cleveland, OH USA; 2 Department of Translational Hematology and Oncology Research, Taussig Cancer Institute, Cleveland Clinic, Cleveland, OH USA

## Abstract

Intermediate-risk acute myeloid leukemia (IR-AML) is a clinically heterogeneous disease, for which optimal post-remission therapy is debated. The utility of next-generation sequencing information in decision making for IR-AML has yet to be elucidated. We retrospectively studied 100 IR-AML patients, defined by European Leukemia Net classification, who had mutational information at diagnosis, received intensive chemotherapy and achieved complete remission (CR) at Cleveland Clinic (CC). The Cancer Genome Atlas (TCGA) data were used for validation. In the CC cohort, median age was 58.5 years, 64% had normal cytogenetics, and 31% required >1 induction cycles to achieve CR1. In univariable analysis, patients carrying mutations in *DNMT3A*, *U2AF1*, and *EZH2* had worse overall and relapse-free survival. After adjusting for other variables, the presence of these mutations maintained an independent effect on survival in both CC and TCGA cohorts. Patients who did not have the mutations and underwent hematopoietic cell transplant (HCT) had the best outcomes. HCT improved outcomes for patients who had these mutations. *RUNX1* or *ASXL1* mutations did not predict survival, and performance of HCT did not confer a significant survival benefit. Our results provide evidence of clinical utility in considering mutation screening to stratify IR-AML patients after CR1 to guide therapeutic decisions.

## Introduction

Acute myeloid leukemia (AML) is a heterogeneous disease characterized by impaired differentiation and increased proliferation of myeloid progenitors. With the widespread use of high-throughput sequencing techniques, AML has been genetically characterized as a complex polyclonal disease with multiple somatically acquired driver mutations and disease evolution over time^1^. In 2013, the Cancer Genome Atlas (TCGA) Research Network profiled the genomes of 200 adult de novo AML patients and identified 23 commonly mutated genes, which were classified into 9 categories^2^. Subsequently, a more comprehensive study of the driver mutation landscape in AML enabled a full genomic classification with nonoverlapping subgroups^3^. These and multiple other studies have yielded important prognostic information and has led cytogenetically normal AML with *NPM1* or biallelic *CEBPA* mutations in the absence of *FLT3*-ITD to be placed in a favorable risk category^4^. Moreover, patients with *RUNX1*, *ASXL1*, and *TP53* mutations have recently been added to the adverse risk group in the European Leukemia Net (ELN) 2017 classification^5^.

Despite the increase in our knowledge of the biology of AML, treatment algorithms have not changed substantially over the last 40 years^6^. Regardless of their mutations and risk stratification, the majority of eligible patients receive intensive induction chemotherapy, with a primary goal of achieving complete remission (CR). This is followed by post-remission therapy tailored according to risk profile, whereby chemotherapeutic consolidation is preferred in favorable-risk AML and allogeneic hematopoietic cell transplant (HCT) is favored in poor-risk AML^5^. The optimal post-remission therapy for intermediate-risk AML, which comprises nearly half of all cases, is debated. Many are evaluated for HCT at the time of CR1^7^, with reduced intensity conditioning (RIC) approaches now available for use in older populations or those with co-morbidities^8–11^. The prognostic role of certain mutations within intermediate-risk AML is unclear, but might aid in decision making for this clinically heterogeneous patient population.

The use of mutational data to inform clinical practice is an active area of research. In a large study of 664 AML patients treated on two phase 3 trials conducted by the German AML Cooperative Group, mutations in *RUNX1*, *SRSF2*, *U2AF1*, and *SF3B1* were found to be independent risk factors for achievement of CR1^12^. In addition, multivariable analysis of a smaller cohort of intermediate-risk AML patients (defined by cytogenetics alone) identified *ASXL1* and *FLT3-ITD* as factors predicting lower chances of achieving CR1^13^. These mutations, along with a few others (e.g., *DNMT3A*, *TP53*) are known to be associated with poor overall survival (OS)^14,15^. However, whether these mutations retain their predictive values after achievement of CR1, at which time the clones harboring these mutations might have already been eradicated, is unknown. Furthermore, the utility of this genetic information in decision making for intermediate-risk AML patients who have achieved CR1 has not been determined. Therefore, we set out to investigate the clinical relevance of recurrent driver gene mutations in a well-characterized cohort of intermediate-risk AML patients who were homogenously treated at a single center and achieved CR1. The analysis aims to re-visit the predictors of outcome at CR1, and identify mutations that portend poor prognosis, hence may help select patients who may benefit from HCT.

## Methods

### Patient cohorts and study eligibility

#### Cleveland clinic (CC) cohort

The diagnosis of AML was made or revised according to the 2016 WHO criteria^16^. Cytogenetic analysis was performed on metaphases from bone marrow aspirates taken at diagnosis, and the risk was ascribed by ELN 2010 criteria as intermediate-I or -II^17^. Namely, patients who had normal cytogenetics and harbored *NPM1* or biallelic *CEBPA* mutations in the absence of *FLT3-ITD* were excluded. A total of 1589 AML patients treated at the Cleveland Clinic between 2002 and 2016 were screened for eligibility, of whom 825 were in ELN intermediate-risk category, and 355 received intensive induction chemotherapy. We retrospectively analyzed 100 intermediate-risk AML patients who had available pre-treatment myeloid mutational data and achieved CR1 after intensive chemotherapy. Patients who did not receive intensive therapy or achieve CR after 1 or more lines of induction (i.e., primary refractory) were excluded.

The induction regimen was 7 + 3 (i.e., 7 days of 100 mg/m^2^ cytarabine plus 3 days of anthracycline), and patients who had persistent disease at day 14 marrow were re-induced with 7 + 3 or 5 + 2 (i.e., 5 days of cytarabine plus 2 days of anthracycline). All patients achieved CR1 with or without count recovery. CR was defined as less than 5% bone marrow blasts with evidence of normal maturation of other marrow elements, no peripheral blast cells or extramedullary disease, peripheral blood neutrophil counts above 1 × 10^9^/L, and platelet counts above 100 × 10^9^/L. Complete response with inadequate count recovery (CRi) was defined as a response meeting the criteria of CR, except for residual neutropenia and/or thrombocytopenia^5^. Induction therapy was followed by consolidation chemotherapy (i.e., high or intermediate dose cytarabine) or allogeneic HCT. Clinical data and patient samples were collected prospectively with patient consent and approved by the Institutional Review Board in accordance with the Declaration of Helsinki.

#### TCGA cohort

A large publicly available database of 200 clinically annotated adult de novo AML patients with genomic and epigenomic profiling was created in 2013^2^. The study analyzed patients from a single institution tissue banking protocol in Washington University and samples were selected to represent morphologic and cytogenetic subtypes of AML. For external validation of our findings, we studied 48 ELN intermediate-risk AML patients from this cohort, who received intensive induction chemotherapy and achieved CR1. Patients who were unfit for intensive chemotherapy, and those with primary refractory disease and acute promyelocytic leukemia were excluded.

### Sample processing, DNA sequencing and mutation analysis

Multi-amplicon deep sequencing was performed as previously described^18^. DNA was extracted from bone marrow or peripheral blood mononuclear cells collected at the time of diagnosis. In 10 patients, subsequent serial samples were also studied. A TruSeq Custom Amplicon panel (TSCA; Illumina, San Diego, CA, USA) targeting coding exons of 62 genes with available evidence in myeloid neoplasms was used for deep sequencing (Supplementary Table). For germline confirmation, mutations were analyzed in non-clonal CD3 positive T cells whenever DNA was available. Bidirectional sequencing was performed by standard techniques using an ABI 3730xl DNA analyzer (Applied Biosystems, Foster City, CA, USA). GATK3.3 pipeline was used to extract putative variants, following recommended best practices for variant discovery. Variants with at least 10 positive reads and variant allelic frequency of 5% were prioritized for further processing and annotation. Generated VCF files were used as an input for Annovar and were annotated with multiple databases (dbSNP138, COSMIC, ExacDb). Variants found in ExacDb with allelic frequency >0.0001 were excluded. Variant allelic frequencies (VAFs) of mutations were adjusted according to the zygosity and copy number confirmed by single nucleotide polymorphism (SNP)-array. In addition, mutations in *NPM1*, *FLT3*, and *CEBPA* were also tested using standard methods. The sequencing method for patients in TCGA database is described previously^2^.

### Statistical analysis

Data were presented with percentage proportions for categorical variables and medians for continuous variables. Comparison of the distribution of categorical variables was carried out with either *χ*^2^-test or Fisher’s exact test. Comparison of numerical variables between groups was performed using Wilcoxon rank sum test. Whisker plot boxes denote median and 25th and 75th percentiles, and ends of the whiskers display minimum and maximum values. Cox proportional hazards regression was used for univariable and multivariable analysis to identify the impact of clinical and genomic variables on survival outcomes. Data were presented with hazard ratios (HR) and 95% confidence intervals (CI). Overall survival (OS) was defined as the time from diagnosis until death or the last follow-up. Patients who were alive were censored at the last follow-up date. Relapse-free survival (RFS) was calculated from the time point of CR1 until the time of relapse or death or the last follow-up. Patients without relapse or death at last follow-up were censored. The survival analysis was based on Kaplan–Meier method and the log-rank test was used to compare the curves. *P* values were two-tailed and considered significant when <0.05. All analyses were performed using JMP software v.12.2.0 (SAS Inc. Cary, NC, USA).

## Results

### Patient characteristics

Clinical characteristics of patients are summarized in Table 1. In the CC cohort, the median age was 58.5 years (range, 24 to 75 years). Forty-eight percent were female, 76% had de novo AML, 19% had secondary AML after an antecedent hematologic disorder (sAML), and 5% had treatment-related AML (tAML). Since all patients in the TCGA cohort had de novo AML, there were more patients with ELN intermediate-I risk category (i.e., normal cytogenetics) as compared to the CC cohort (81 vs 64%, *p* = 0.007). Concomitantly, patients in the TCGA cohort had a higher WBC count and bone marrow blast percentage at diagnosis. In the CC cohort, initial 7 + 3 induction chemotherapy resulted in CR in 58%, CRi in 11%, and persistent disease in 31% of patients based on day 14 bone marrow biopsy. Of the 31 patients with persistent disease, 20 were re-induced with 5 + 2, and 11 received 7 + 3. Best response achieved was CR in 23 patients and CRi in 8 patients. The number of patients who received HCT and the time of HCT were not significantly different between cohorts. However, there were more patients who underwent haploidentical HCT in our cohort, and 13% of the transplanted patients in the TCGA cohort received autologous HCT.

**Table 1:** Characteristics of AML patients who achieved CR1 after intensive chemotherapy

Variable	CC cohort (*N* = 100)	TCGA cohort (*N* = 48)	*P* value
*Age*			
Median, years (range)	58.5 (24–75)	57 (22–77)	0.61
<60 years, %	55	60.4	
≥60 years, %	45	40.6	
Female, %	48	42	0.39
*AML type, %*			
De novo	76	100	<0.001
Secondary	19	0	
Treatment-related	5	0	
*WBC count* *(* *×10* ^*9*^ */L)*			
Median, range	5.6 (0.55–241)	30.1 (0.6–223.8)	0.001
*Bone marrow blasts, %*			
Median, range	53 (6–95)	75 (34–100)	<0.001
*Extramedullary disease, %*			
Skin	4	NA	
CNS	2		
Lymph node	1		
*ELN 2010 classification, %*			
Intermediate-I	64	81	0.007^a^
Intermediate-II	36	19	
Trisomy 8	7	8.3	
MLLT3-MLL present	4	0	
*Response to first induction* ^*b*^ *,* *%*			
CR	58	NA	
CRi	11		
Persistent disease	31		
*Number of induction courses to achieve CR1*			
1	69	NA	
≥2	31		
*Time to relapse*			
Median, range	21 (2–70)	12 (1.7–69)	0.16
*Time of HCT, %*			
In CR1	37	48	0.07
In CR2	13	10	
At first relapse	5	0	
No HCT	45	42	
*Type of HCT, %*			
MSD	41.8	53.4	0.0001
MUD	38.2	30	
Haploidentical	18.2	3.3	
Auto	0	13.3	
UCB	1.8	0	
*Conditioning regimen*			
Myeloablative	26	NA	
BuCy	21		
FluTBI	3		
Other	2		
Reduced-intensity	29		
BuFlu	19		
FluCyTBI	6		
Other	4		

*AML* acute myeloid leukemia, *Bu* busulfan, *CC* Cleveland Clinic, *CNS* central nervous system, *CR* complete response, *CRi* CR with incomplete count recovery, *Cy* cyclophosphamide, *Flu* fludarabine, *HCT* hematopoietic cell transplant, *MSD* matched sibling donor, *MUD* matched unrelated donor, *NA* not available, *TBI* total body irradiation, *UCB* umbilical cord blood, *WBC* white blood cell

^a^*P* value compares the percentages of patients in intermediate-I risk category

^b^Response to first induction was determined by day 14 bone marrow biopsy

The median OS and RFS of the CC cohort were 24 (range, 2 to 108 months) and 14 months (range, 1 to 70 months), respectively. Sixty patients remained in remission after a median follow-up of 14.5 months (range, 2 to 108 months). The survival outcomes of the TCGA cohort were similar to ours with a median OS of 24.4 (range, 2.2 to 118.1 months) and median RFS of 13.4 months (range, 1.7 to 77.3 months).

### Spectrum of driver mutations

**Fig. 1:**
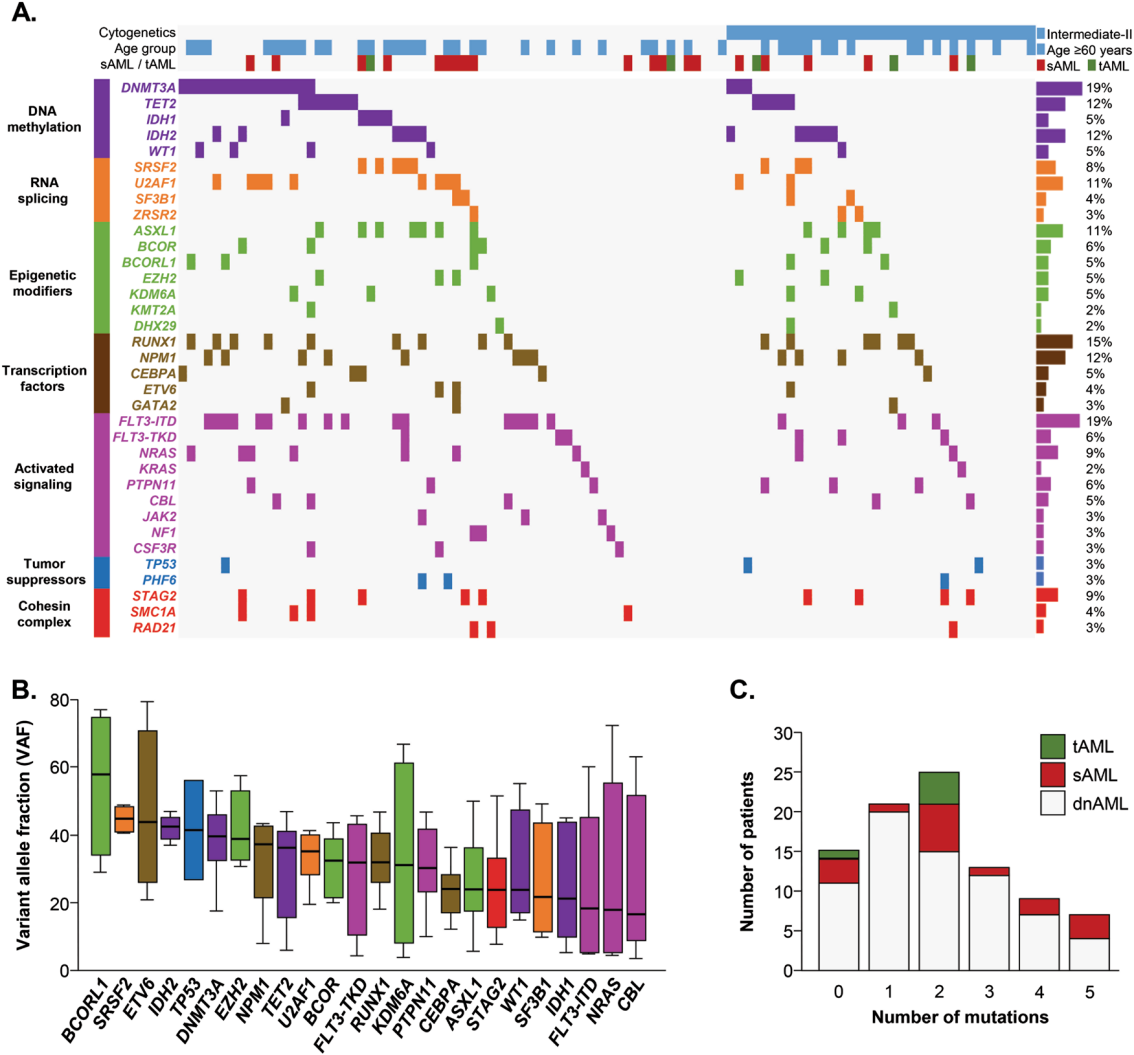
Overview of driver gene mutations identified by targeted sequencing in 100 AML patients **a** Heatmap depicting gene mutations in 100 adult AML patients in the Cleveland Clinic cohort. **b** Analysis of variant allelic frequencies (VAFs) of driver gene mutations found in ≥5 patients. **c** Number of driver gene mutations in patients with de novo AML (dnAML) compared with secondary AML (sAML) and treatment-related AML (tAML)

The mutational spectrum of our cohort is illustrated in Fig. 1. *DNMT3A* and *FLT3-ITD* mutations were the most common (19%), followed by *RUNX1* (15%), *TET2* (12%), *IDH2* (12%), *NPM1* (12%), *ASXL1* (11%), and *U2AF1* (11%) (Fig. 1a). At least one driver mutation was identified in 85% of patients. Of the remaining 15 patients, 6 had cytogenetic abnormalities. Thus, ≥1 molecularly defined lesion was detected in 91% of cases. The median number of driver gene mutations per patient was 2 (range, 0–12), and the median VAF of mutations was 33.3% (range, 5–94.3%). The spectrum of VAFs observed for genes mutated in ≥5 patients are illustrated in Fig. 1b. Of note, three genes with the lowest median VAFs (FLT3, *NRAS*, and *CBL*) are involved in growth factor signaling, supporting the previous observations that alterations in these pathways are acquired later during the evolution of the leukemic clone^12,19^.

We studied the association of driver gene mutations with clinical and cytogenetic characteristics. The median number of mutations was higher in patients aged ≥ 60 years as compared to younger patients (3 vs 2, *p* = 0.03), and these patients more commonly carried mutations in *SRSF2* (16% vs 2%, *p* = 0.02) and *NRAS* (16% vs 4%, *p* = 0.03). Mutations in *ASXL1* and *SRSF2* were more common in men compared to women (19% vs 2%, *p* = 0.005 and 13% vs 2%, *p* = 0.03, respectively). The number of mutated genes was not significantly different between de novo AML, sAML, and tAML (Fig. 1c). However, *FLT3-ITD* and *NPM1* mutations were seen exclusively in de novo AML (*p* = 0.003 and *p* = 0.03, respectively). Patients with normal cytogenetics had higher rates of *DNMT3A* (25 vs 8%, *p* = 0.03) and *FLT3-ITD* (24 vs 8%, *p* = 0.04) mutations than patients who had abnormal karyotype. Finally, the frequency of persistent disease after initial induction chemotherapy was higher in patients with mutations in *DNMT3A* (47 vs 27%, *p* = 0.03) and *U2AF1* (55 vs 28%, *p* = 0.01), while CRi was more common in patients with *BCOR* mutation (50% vs 8.5%, *p* = 0.02). Of note, all of the 6 patients with *FLT3-TKD* achieved CR after first induction (100% vs 55.3%, *p* = 0.03).

### Clinical and genetic predictive features for outcomes after CR1 in intermediate-risk AML

We analyzed the impact of several prognostic variables on OS and RFS after CR1 in our cohort of intermediate-risk AML patients. In univariable analysis (Table 2), patients who achieved CRi after induction chemotherapy had a higher risk of death (HR, 2.4; 95% CI, 1.22–4.46; *p* = 0.01) and relapse (HR, 2.04; 95% CI, 1.1–3.6; *p* = 0.02) as compared to patients who achieved CR. Furthermore, patients who underwent HCT achieved better OS and RFS, represented by the unadjusted risk of 0.4 (95% CI, 0.22–0.72; *p* = 0.003) for death, and 0.34 (95% CI, 0.18–0.6; *p* = 0.0002) for relapse. Other clinical factors analyzed, such as age, sex, de novo vs. sAML/tAML, WBC count, extramedullary disease, cytogenetics, and the number of induction courses to achieve CR1 were not significant predictors for survival after CR1. Additionally, we could not demonstrate a difference in outcome when various donor sources and the intensity of conditioning regimen for HCT were compared.

**Table 2:** Univariable analysis of clinical variables and myeloid mutations in the Cleveland Clinic cohort

	Overall survival, months		Relapse-free survival, months	
	HR (95% CI)	*p*	HR (95% CI)	*p*
Age (continuous)	1.02 (0.99–1.05)	0.18	1 (0.98–1.03)	0.84
≥60 v <60 years	1.39 (0.78–2.47)	0.26	1.15 (0.67–1.92)	0.6
Sex (female v male)	1.07 (0.6–1.9)	0.82	0.96 (0.57–1.61)	0.88
*AML type*				
Secondary v de novo	1.65 (0.79–3.16)	0.17	1.19 (0.6–2.18)	0.61
Therapy-related v de novo	0.64 (0.1–2.11)	0.51	0.6 (0.1–1.94)	0.44
WBC count (x10^9^/L) (continuous)	1 (0.99–1.01)	0.51	1 (0.99–1.01)	0.87
Extramedullary disease (yes v no)	1.31 (0.32–3.62)	0.66	1.03 (0.25–2.81)	0.96
*Cytogenetics*				
Intermediate-II v -I	0.99 (0.54–1.77)	0.97	1.17 (0.68–1.96)	0.57
*Number of induction courses to achieve CR1*				
≥2 v 1	1.34 (0.73–2.39)	0.34	1.21 (0.69–2.05)	0.5
*Best response to induction/re-induction therapy*				
CRi v CR	2.4 (1.22–4.46)	0.01	2.04 (1.1–3.6)	0.02
HCT (yes v no)	0.4 (0.22–0.72)	0.003	0.34 (0.18–0.6)	0.0002
*Type of HCT*				
MUD v MSD	1.03 (0.39–2.6)	0.96	0.76 (0.2–2.53)	0.66
Haploidentical v MSD	2.55 (0.85–7.05)	0.09	1.68 (0.35–6.24)	0.48
*Conditioning regimen*				
RIC v MAC	1.08 (0.48–2.47)	0.85	1.03 (0.35–3.01)	0.96
*Myeloid mutations*				
*FLT3-ITD*	1.05 (0.48–2.08)	0.89	0.93 (0.44–1.76)	0.83
*DNMT3A*	2.46 (1.16–4.86)	0.02	1.9 (0.95–3.37)	0.06
*RUNX1*	0.99 (0.41–2.08)	0.98	1.09 (0.52–2.06)	0.81
*TET2*	0.98 (0.34–2.27)	0.97	0.83 (0.29–1.89)	0.69
*IDH2*	1.35 (0.55–2.83)	0.48	1.19 (0.52–2.37)	0.66
*NPM1*	0.79 (0.24–1.96)	0.64	0.74 (0.26–1.69)	0.5
*U2AF1*	3.61 (1.61–7.31)	0.003	2.37 (1.08–4.64)	0.03
*ASXL1*	1.3 (0.49–2.85)	0.56	1.45 (0.63–2.91)	0.36
*NRAS*	2.59 (0.97–5.74)	0.06	1.49 (0.57–3.23)	0.38
*STAG2*	1.21 (0.41–2.82)	0.7	0.95 (0.33–2.17)	0.9
*SRSF2*	2.28 (0.67–5.83)	0.16	1.24 (0.37–3.07)	0.69
*FLT3-TKD*	0.37 (0.02–1.7)	0.25	0.53 (0.09–1.7)	0.33
*PTPN11*	0.56 (0.09–1.81)	0.38	0.91 (0.28–2.24)	0.86
*BCOR*	1.4 (0.34–3.88)	0.59	1.78 (0.54–4.4)	0.31
*BCORL1*	1.78 (0.43–4.92)	0.37	1.83 (0.55–4.5)	0.29
*EZH2*	3.75 (0.88–10.98)	0.06	3.26 (0.77–9.46)	0.09
*IDH1*	1.25 (0.3–3.44)	0.72	1 (0.24–2.72)	0.99
*CEBPA* (single)	0.53 (0.23–2.43)	0.49	0.39 (0.02–1.79)	0.28
*WT1*	0.37 (0.02–1.69)	0.25	1.06 (0.26–2.88)	0.93
*KDM6A*	1.5 (0.36–4.14)	0.52	1.59 (0.39–4.34)	0.47
*CBL*	1.38 (0.33–3.8)	0.61	1.5 (0.45–3.69)	0.46

*AML* acute myeloid leukemia, *CI* confidence interval, *CR* complete response, *CRi* CR with incomplete count recovery, *HCT* hematopoietic cell transplant, *HR* hazard ratio, *MAC* myeloablative conditioning, *MSD* matched sibling donor, *MUD* matched unrelated donor, *RIC* reduced intensity conditioning, *WBC* white blood cell

**Fig. 2:**
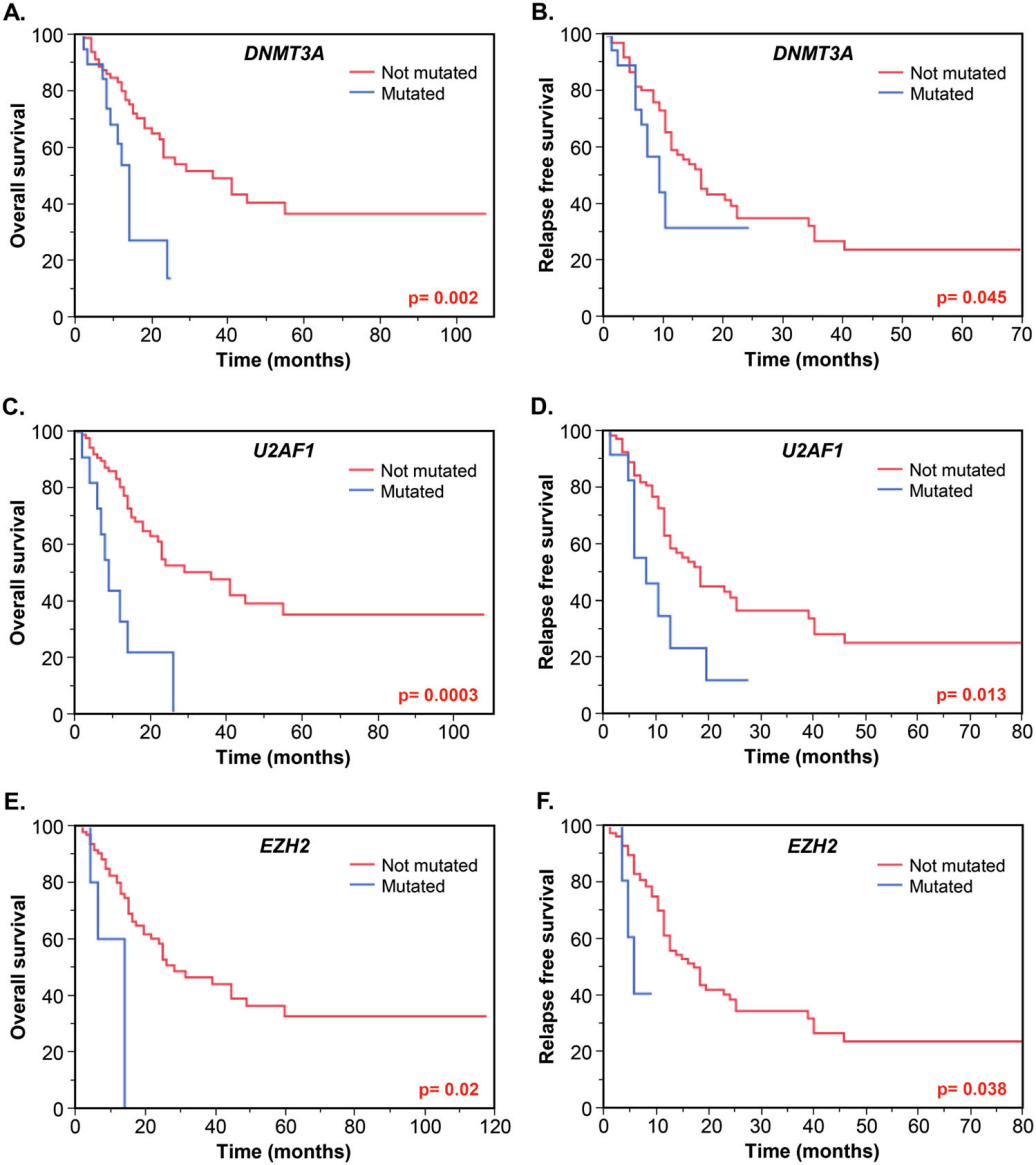
Kaplan–Meier overall survival (OS) and relapse-free survival (RFS) curves of intermediate-risk AML patients stratified based on their mutations in *DNMT3A* **a**,** b**, *U2AF1* **c**,** d**, and *EZH2* **e**,** f** in the Cleveland Clinic cohort

Univariable analyses of gene mutations demonstrated that survival was significantly shorter for patients carrying mutations in *DNMT3A*, *U2AF1*, and *EZH2* (Fig. 2). The median OS and RFS for *DNMT3A* mutated patients were 12 and 9 months respectively, as compared to 35 and 16 months in patients without mutation (*p* = 0.002 and 0.045; Fig. 2a, b). The presence of a *DNMT3A* mutation conferred an unadjusted risk of 2.46 (95% CI, 1.16–4.86; *p* = 0.02) for death, and 1.9 (95% CI, 0.95–3.37; *p* = 0.06) for relapse (Table 2). Patients with *U2AF1* mutation had significantly worse OS (median, 9 vs 36 months, *p* = 0.0003) and RFS (median, 7 vs 16 months, *p* = 0.013) than patients without mutation (Fig. 2c, d), with an unadjusted risk of 3.61 (95% CI, 1.61–7.31; *p* = 0.003) for death, and 2.37 (95% CI, 1.08–4.64; *p* = 0.03) for relapse. Finally, the *EZH2* mutation was associated with significantly shorter OS (median, 13 vs 26 months, *p* = 0.02) and RFS (median, 5 vs 15 months, *p* = 0.038) (Fig. 2e, f), with unadjusted risk of 3.75 (95% CI, 0.88–10.98; *p* = 0.06) and 3.26 (95% CI, 0.77–9.46; *p* = 0.09), respectively.

**Fig. 3:**
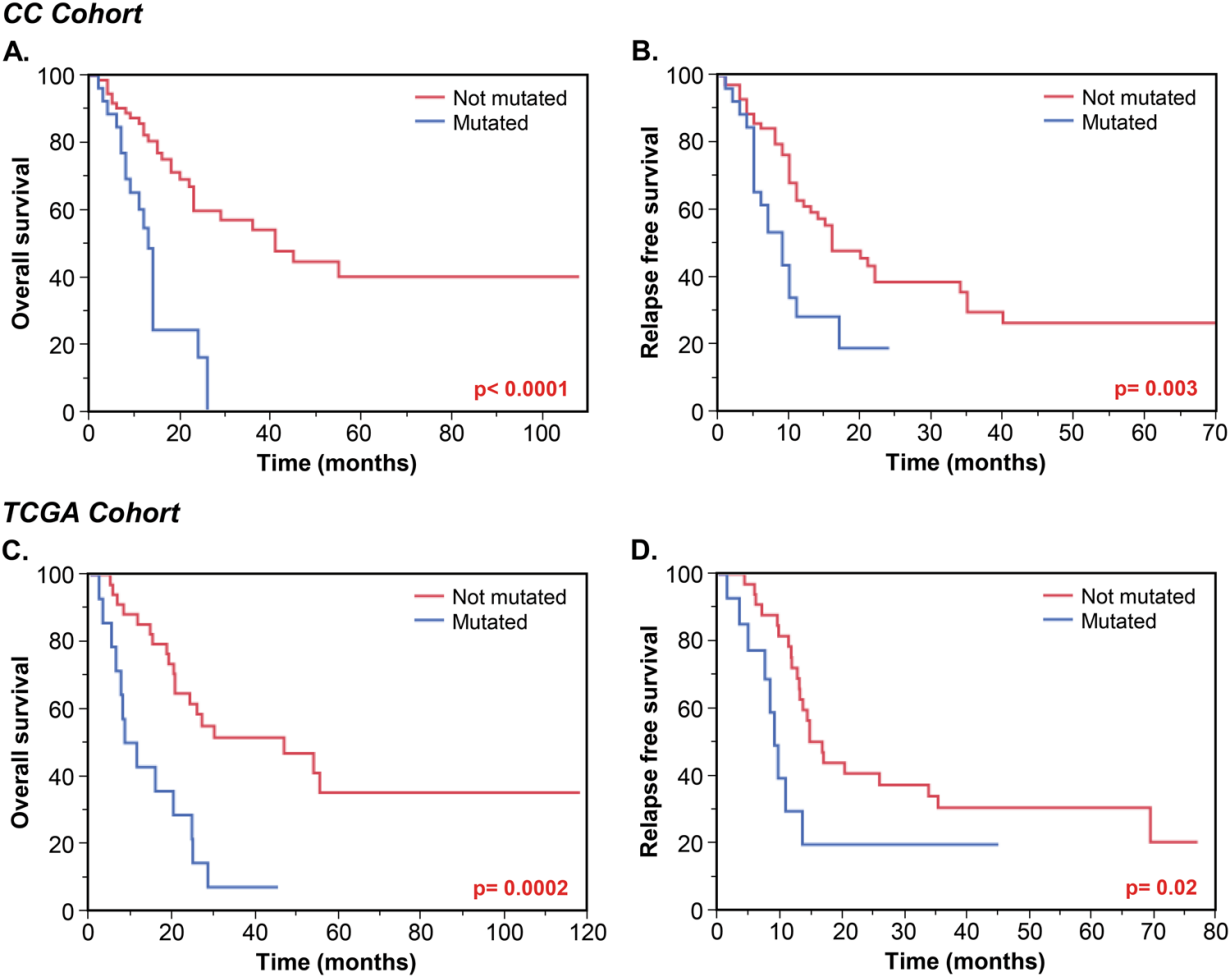
Kaplan–Meier overall survival (OS) and relapse-free survival (RFS) curves of intermediate-risk AML patients stratified based on their mutations in *DNMT3A, U2AF1*, and *EZH2* in the Cleveland Clinic (CC) **a**,** b** and the Cancer Genome Atlas (TCGA) cohorts **c**,** d**

To verify whether the presence of these three mutations could improve the prognostic stratification of patients with intermediate-risk AML at CR1, we compared CC patients who harbored any of the three mutations with those who did not (Fig. 3). The risk stratification for OS was improved with the combined model as compared to the use of single mutations (Fig. 3a). The median OS for patients with mutations was 13 months, as compared to 41 months in patients without them (*p* < 0.0001). The median RFS was 9 vs 16 months (*p* = 0.003) (Fig. 3b). Similarly, when the mutation-based stratification was applied to intermediate-risk patients in the TCGA cohort, mutated patients had significantly poorer OS (median, 9.8 vs 46.8 months, *p* = 0.0002) and RFS (median, 9.3 vs 17 months, *p* = 0.02) (Fig. 3c, d). Moreover, after adjusting for the type of response (CR/CRi) and HCT status in multivariable analysis, the presence of the mutations maintained an independent effect on OS and RFS (HR, 3.22; 95% CI, 1.66–6.16; *p* = 0.0007 and HR, 2.17; 95%CI, 1.78–3.87; *p* = 0.01, respectively) (Table 3).

**Table 3:** Multivariable analysis for overall and relapse-free survival in the Cleveland Clinic cohort

	Overall survival, months		Relapse-free survival, months	
	HR (95% CI)	*P*	HR (95% CI)	*P*
*Best response to induction/re-induction therapy*				
CRi v CR	2.49 (1.23–4.79)	0.01	2.26 (1.19–4.14)	0.01
HCT (yes v no)	0.37 (0.2–0.69)	0.002	0.47 (0.27–0.82)	0.009
Mutated v not mutated	3.22 (1.66–6.16)	0.0007	2.17 (1.78–3.87)	0.01

*CI* confidence interval, *CR* complete response, *CRi* CR with incomplete count recovery, *HCT* hematopoietic cell transplant, *HR* hazard ratio

### Outcomes of patients with *DNMT3A*, *U2AF1*, and *EZH2* mutations based on the performance of HCT

**Fig. 4:**
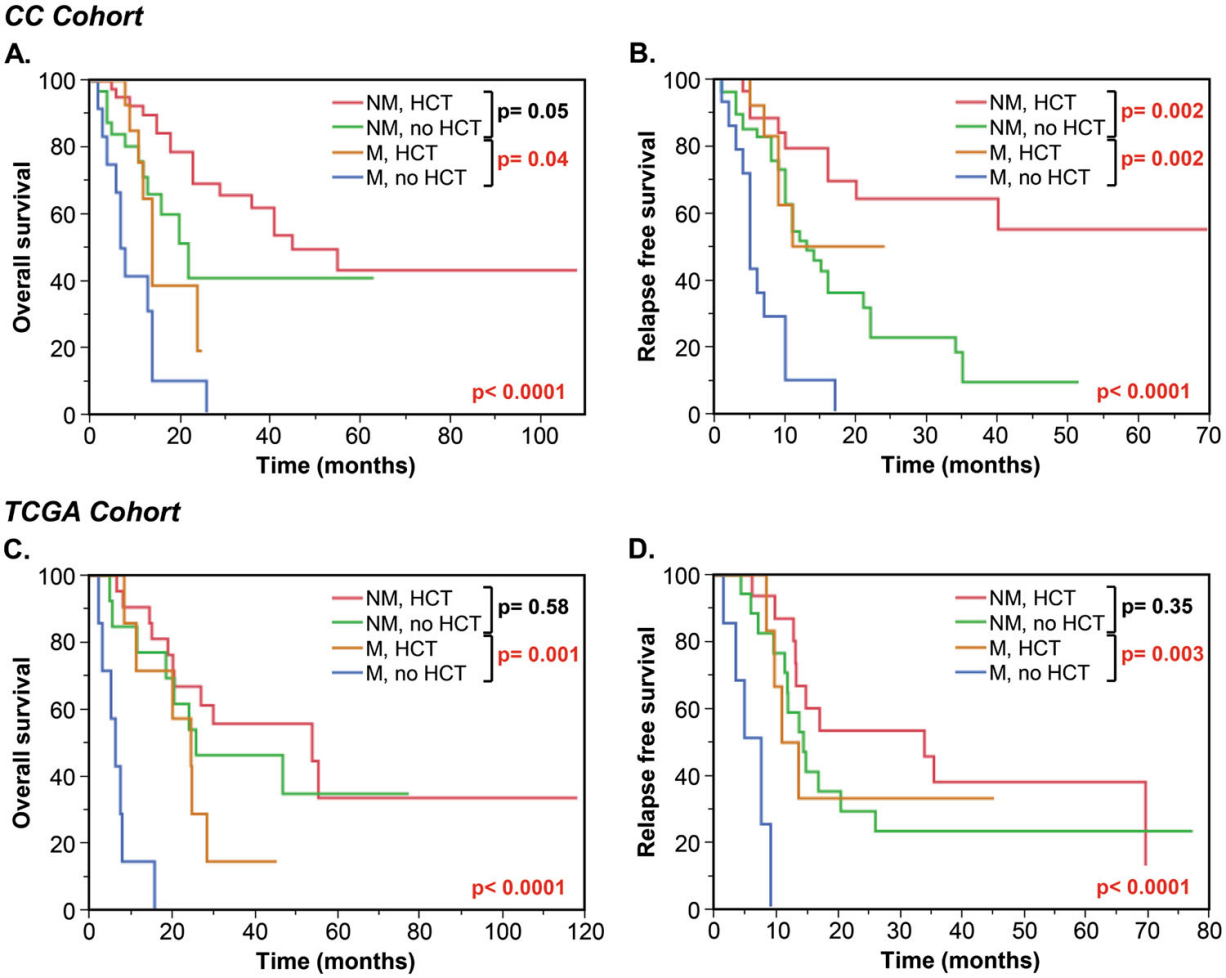
Kaplan–Meier overall survival (OS) and relapse-free survival (RFS) curves of intermediate-risk AML patients, stratified based on the presence of mutations in *DNMT3A, U2AF1*, and *EZH2*, and performance of hematopoietic cell transplant (HCT) in the Cleveland Clinic (CC) **a**,** b** and the Cancer Genome Atlas (TCGA) cohorts **c**,** d**

Based on the finding that mutations and HCT were the major predictors of outcome after CR1, we analyzed the outcomes of patients harboring mutations in relation to HCT (Fig. 4). In both CC and TCGA cohorts, patients who did not have the mutations and underwent HCT had the best outcomes. In the CC cohort, the median OS and RFS for these patients were 45 months and not reached, respectively, as compared to 22 and 13 months for patients who did not undergo HCT (*p* = 0.05 for OS and *p* = 0.002 for RFS). In the TCGA cohort, performance of HCT did not have a significant impact on OS and RFS in patients without these mutations. However, HCT improved outcomes for patients who had these mutations in both cohorts. In the CC cohort, median OS and RFS for these patients who had HCT were 14 and 11 months, respectively, as compared to 7.5 and 5 months in patients who did not undergo HCT (*p* = 0.04 for OS and *p* = 0.002 for RFS). In the TCGA cohort, patients who harbored mutations and did not undergo HCT had significantly worse OS (median, 6.3 vs 24.6 months, *p* = 0.001) and RFS (median, 7.8 vs 12.5 months, *p* = 0.003), while there were no statistically significant differences between survival of patients with mutations who underwent HCT and patients who did not have the mutations.

In view of the recently proposed ELN 2017 criteria that place intermediate-risk patients with *RUNX1* or *ASXL1* mutations into the adverse risk category, which leads to the recommendation of HCT for this group, we explored outcomes of these patients at the time of CR1 (Supplementary Figure 1). Presence of these mutations did not predict OS and RFS in our cohort, and performance of HCT in mutated patients did not confer a statistically significant survival benefit.

### Driver mutation dynamics and clinical course

**Fig. 5:**
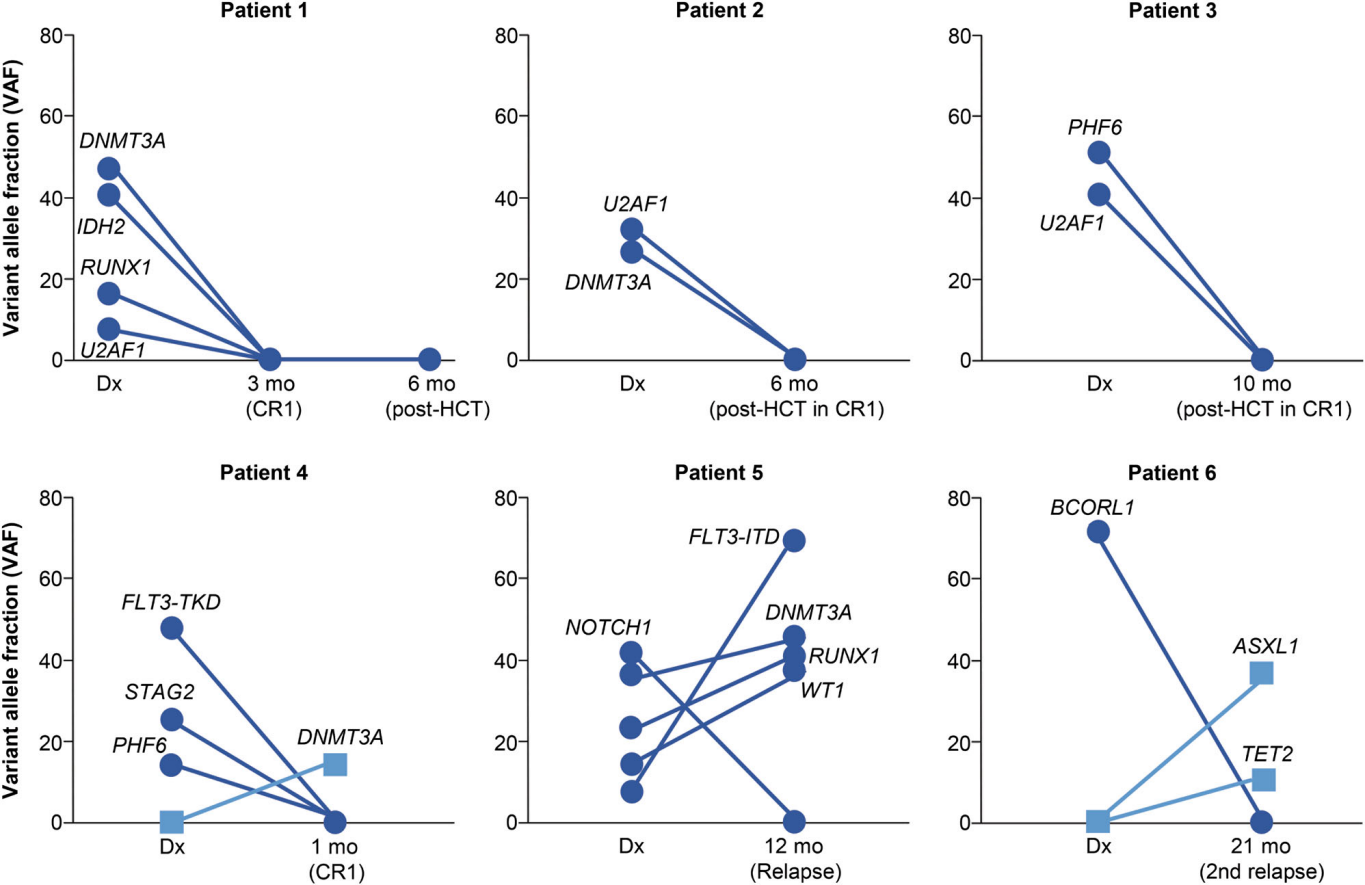
Dynamics of major driver mutations revealed by targeted sequencing and graphed with time charts showing changes in their variant allelic frequencies (VAFs) during disease progression in six representative patients

In light of the above results, we next evaluated the serially collected samples from 10 patients (Fig. 5). The serial sequencing data reveal interesting and informative aspects about the clonal evolution of AML. The majority of patients harbored >1 clone at diagnosis, which were eradicated with intensive therapy. For example, patient 1 had *DNMT3A* and *IDH2* mutations at VAF > 40%, which disappeared after 7 + 3. The patient underwent HCT in CR1 and was disease-free at 25 months follow-up. Patients 2 and 3 had *U2AF1* mutated clones, which were absent following HCT in CR1. However, both of them relapsed shortly after HCT and died at 12 and 14 months follow-up, respectively. Additionally, patient 4 harbored three different mutations, which did not predict survival in this study, and they were eradicated with induction chemotherapy. However, the patient had evidence of an emerging *DNMT3A* clone, and relapsed 9 months after achieving CR1. Of interest, patient 5 had clones with *FLT3-ITD*, *DNMT3A*, *RUNX1*, and *WT1* mutations, which persisted after chemotherapy and increased in size at the time of relapse. This patient died 2 months after the second sampling. Finally, patient 6 had a dominant *BCORL1* clone, which disappeared with therapy, and relapsed with the emergence of new *ASXL1* and *TET2* mutations. The patient achieved CR3 after relapse therapy and was disease-free at 40 months follow-up. The evidence of clonal evolution for additional three patients is summarized in Supplementary Figure 2. We could not demonstrate driver mutations at diagnosis and during follow-up in one patient.

## Discussion

The optimal post-remission consolidation therapy for intermediate-risk AML has been debated for years due to the clinical heterogeneity in outcomes of this group. These patients were sub-categorized based on normal vs abnormal cytogenetics (e.g., trisomy 8, *MLLT3-MLL* rearrangement) as intermediate-I and -II, respectively, but subsequent studies demonstrated no survival difference in between, which has led to the lumping of two groups in the recently proposed classification^5,20^. Considering the insufficiency of cytogenetic data to distinguish the differences in outcomes of this group, we hypothesized that driver mutations may have an effect in predicting outcomes of intermediate-risk AML and aid in clinical decision making with a particular focus on performance of HCT. Since achieving CR after intensive induction is a prerequisite for long-term survival in AML and 1-year mortality rate is >75% for primary refractory cases^21^, the present study focused on patients who achieved CR with 1 or more induction courses. This strategy enabled a more accurate assessment of the impact of HCT vs consolidation with chemotherapy on outcomes of patients stratified by mutational status. In two independent cohorts, we demonstrated that mutations in *DNMT3A*, *U2AF1*, and *EZH2* genes were independent predictors of relapse and OS in patients who achieved CR, and performance of HCT in this group translated into a significant survival benefit.

One major difference between the CC and TCGA cohorts was that all patients in the latter had de novo AML, which accounted for higher WBC count, bone marrow blast percentage, and percentage of patients with normal cytogenetics in TCGA cohort. Donor sources were also slightly different for transplanted patients. However, none of these differences were found to predict survival in our univariable analysis. The cohorts were otherwise similar in terms of demographics, numbers of transplanted patients, and median survival durations.

The prognostic significance of *DNMT3A* and *U2AF1* mutations in AML has been shown in a few other studies^12,22,23^. *DNMT3A* mutations in patients <60 years and *U2AF1* mutations were independently associated with a lower CR rate after induction therapy^12^. In a large cohort of AML patients reported by Ley, et al.^15^, *DNMT3A* mutation was an independent predictor of poor survival in intermediate-risk cytogenetics group. Additionally, *EZH2* inactivation was associated with poor prognosis in myelodysplastic syndromes, while its role in AML remains unclear^24^. In this study, patients with mutated *EZH2* had significantly shorter OS and RFS, and a model integrating these three mutations could stratify intermediate-risk AML patients into two risk groups both in the CC and TCGA cohorts. Furthermore, eradication of these clones with induction chemotherapy, followed by HCT led to long-term survival in patients with available serial sequencing data. On the contrary, emergence or persistence of these mutations was associated with worse outcomes. Therefore, assessments of the mutations at diagnosis and during follow-up might offer an opportunity to improve prognostication and management of intermediate-risk AML. While patients with mutations who underwent HCT had improved OS and RFS, the median survival was still shorter than transplanted patients without mutations, indicating the importance of developing further therapies targeting these clones.

Integration of the accumulating information on gene mutations, cytogenetics, and other markers into risk stratification and management algorithms is a critical yet challenging task. Since the demonstration of favorable prognosis associated with *NPM1* and biallelic *CEBPA* mutations, they have been incorporated into prognostic models and routinely tested in all patients at the time of diagnosis^4,17^. HCT is no longer recommended at CR1 for patients with these mutations, who were once classified in intermediate-risk category and transplanted without additional survival benefit. With rigorous efforts towards better risk-stratification in intermediate-risk group, several studies reported adverse outcomes in patients with *RUNX1* and *ASXL1* mutations^25–30^. These reports convinced the ELN panel and led to the re-classification of intermediate-risk patients harboring these two mutations in the poor-risk group^5^. However, when we stratified our cohort based on *RUNX1*/*ASXL1* mutational status, no survival difference was appreciated. More importantly, performance of HCT did not extend survival at a significant level. These results might be attributed to two factors: First, our cohort was selected for patients who achieved CR and mutations in *RUNX1/ASXL1* are known to be associated with refractory disease. Therefore, the poor prognostic impact of these mutations might disappear with the achievement of CR. The lack of survival difference, as well as no survival benefit in transplanted patients, might be due to a relatively lower number of patients enrolled. However, an important biological characteristic reported by multiple studies is that the mutations in *RUNX1* and *ASXL1* tend to co-occur, but are mutually exclusive with mutations in *NPM1* and *CEBPA*^2,3,12^. While previous reports have shown independent prognostic value of *RUNX1* and *ASXL1*, some focused only on cytogenetically normal AML without adjusting for *NPM1* and biallelic *CEBPA* in multivariable models^26,29^, while in other reports, the impact on OS was lost when adjusted for these mutations^25,27,30^. Therefore, the survival differences in these studies are skewed, as *RUNX1/ASXL1* mutated intermediate-risk patients were compared with those who had favorable *NPM1* and *CEBPA* mutations. Based on the results of present study, and highlighted issues in previous reports, the utility of *RUNX1* and *ASXL1* mutations in stratifying intermediate-risk AML is debated and further investigation of HCT with these mutations is warranted.

There are potential limitations in our work, mainly related to the retrospective nature of this study. These include missing sequencing data and samples in a proportion of patients, different types of transplantation and conditioning regimens. Furthermore, in the absence of a matched control sample, distinguishing somatic and germline variants is challenging. Despite these limitations, clinical and molecular data were available in the majority of original patient population, and the results were successfully validated in an external cohort. In addition, the landscape of truly somatic mutations in analyzed genes has been well defined from large-scale genomic studies, which allowed us to make confident predictions. Finally, comparisons of HCT should be treated with caution, since no statistical method can adjust for the unmeasured selection factors involved in a retrospective analysis.

Collectively, our results provide evidence of clinical utility in considering mutation screening to stratify intermediate-risk AML patients after CR1 to guide therapeutic decisions. Mutations in *DMT3A*, *U2AF1*, and *EZH2* might be useful to select patients who would benefit from HCT. On the contrary, *RUNX1* and *ASXL1* mutations were not as useful to predict patients with poor prognosis. A prospective validation of our findings is needed and we believe that our results may contribute to improving prognostication of patients with AML and the design of clinical trials.

## Electronic supplementary material

SUPPLEMENTARY FIGURE LEGENDS

Supplementary table

Supplementary Figure 1

Supplementary Figure 2

## References

[1] Döhner HWD, Bloomfield CD (2015). Acute myeloid leukemia. N. Engl. J. Med..

[2] Network CGAR. (2013). Genomic and epigenomic landscapes of adult de novo acute myeloid leukemia. N. Engl. J. Med..

[3] Papaemmanuil EGM (2016). Genomic classification and prognosis in acute myeloid leukemia. N. Engl. J. Med..

[4] Schlenk RF (2008). Mutations and treatment outcome in cytogenetically normal acute myeloid leukemia. N. Engl. J. Med..

[5] Döhner HEE (2017). Diagnosis and management of AML in adults: 2017 ELN recommendations from an international expert panel. Blood.

[6] Saygin C, Carraway HE (2017). Emerging therapies for acute myeloid leukemia. J. Hematol. Oncol..

[7] Cornelissen JJ, Blaise D (2016). Hematopoietic stem cell transplantation for patients with AML in first complete remission. Blood.

[8] Ferrara F (2012). Renaissance of autologous stem cell transplantation for AML?. Lancet Oncol..

[9] Sengsayadeth S (2015). Reduced intensity conditioning allogeneic hematopoietic cell transplantation for adult acute myeloid leukemia in complete remission - a review from the Acute Leukemia Working Party of the EBMT. Haematologica.

[10] Versluis J (2015). Prediction of non-relapse mortality in recipients of reduced intensity conditioning allogeneic stem cell transplantation with AML in first complete remission. Leukemia.

[11] Cornelissen JJ (2015). Comparative therapeutic value of post-remission approaches in patients with acute myeloid leukemia aged 40-60 years. Leukemia.

[12] Metzeler KH (2016). Spectrum and prognostic relevance of driver gene mutations in acute myeloid leukemia. Blood.

[13] Wang B (2016). Mutational spectrum and risk stratification of intermediate-risk acute myeloid leukemia patients based on next-generation sequencing. Oncotarget.

[14] Patel JP (2012). Prognostic relevance of integrated genetic profiling in acute myeloid leukemia. N. Engl. J. Med..

[15] Ley TJ (2010). DNMT3A mutations in acute myeloid leukemia. N. Engl. J. Med..

[16] Daniel A (2016). The 2016 revision to the World Health Organization classification of myeloid neoplasms and acute leukemia. Blood.

[17] Döhner H (2010). Diagnosis and management of acute myeloid leukemia in adults: recommendations from an international expert panel, on behalf of the European LeukemiaNet. Blood.

[18] Nazha A (2016). Incorporation of molecular data into the Revised International Prognostic Scoring System in treated patients with myelodysplastic syndromes. Leukemia.

[19] Ding L (2012). Clonal evolution in relapsed acute myeloid leukaemia revealed by whole-genome sequencing. Nature.

[20] Mrózek K (2012). Prognostic significance of the European LeukemiaNet standardized system for reporting cytogenetic and molecular alterations in adults with acute myeloid leukemia. J. Clin. Oncol..

[21] Schlenk RF (2003). Risk-adapted postremission therapy in acute myeloid leukemia: results of the German multicenter AML HD93 treatment trial. Leukemia.

[22] Hou HA (2016). Splicing factor mutations predict poor prognosis in patients with de novo acute myeloid leukemia. Oncotarget.

[23] Hamilton BK (2016). Impact of allogeneic hematopoietic cell transplant in patients with myeloid neoplasms carrying spliceosomal mutations. Am. J. Hematol..

[24] Ernst T (2010). Inactivating mutations of the histone methyltransferase gene EZH2 in myeloid disorders. Nat. Genet..

[25] Gaidzik VI (2011). RUNX1 mutations in acute myeloid leukemia: results from a comprehensive genetic and clinical analysis from the AML study group. J. Clin. Oncol..

[26] Mendler JH (2012). RUNX1 mutations are associated with poor outcome in younger and older patients with cytogenetically normal acute myeloid leukemia and with distinct gene and MicroRNA expression signatures. J. Clin. Oncol..

[27] Gaidzik VI (2016). RUNX1 mutations in acute myeloid leukemia are associated with distinct clinico-pathologic and genetic features. Leukemia.

[28] Metzeler KH (2011). ASXL1 mutations identify a high-risk subgroup of older patients with primary cytogenetically normal AML within the ELN Favorable genetic category. Blood.

[29] Schnittger SEC (2013). ASXL1 exon 12 mutations are frequent in AML with intermediate risk karyotype and are independently associated with an adverse outcome. Leukemia.

[30] Paschka P (2015). ASXL1 mutations in younger adult patients with acute myeloid leukemia: a study by the German-Austrian Acute Myeloid Leukemia Study Group. Haematologica.

