# Supplementary material for: Mutations in DNMT3A, U2AF1, and EZH2 identify intermediate-risk acute myeloid leukemia patients with poor outcome after CR1
Source: Blood Cancer J. 2018 Jan 10;8(1):4. doi: 10.1038/s41408-017-0040-9 (PMC5802549; doi:10.1038/s41408-017-0040-9)
Supplement: Supplementary file 1 — SUPPLEMENTARY FIGURE LEGENDS [file 41408_2017_40_MOESM1_ESM.docx]

**SUPPLEMENTARY FIGURE LEGENDS**

**Supplementary Figure 1.** Kaplan-Meier overall survival (OS) and relapse-free survival (RFS) curves of intermediate-risk AML patients, stratified based on the presence of mutations in *RUNX1*, and *ASXL1* **(A, B)**, and performance of hematopoietic cell transplant (HCT) **(C, D)** in the Cleveland Clinic cohort.

**Supplementary Figure 2.** Dynamics of major driver mutations revealed by targeted sequencing and graphed with time charts showing changes in their variant allelic frequencies (VAFs) during disease progression in 3 additional patients.
