# Supplementary material for: Mutations in DNMT3A, U2AF1, and EZH2 identify intermediate-risk acute myeloid leukemia patients with poor outcome after CR1
Source: Blood Cancer J. 2018 Jan 10;8(1):4. doi: 10.1038/s41408-017-0040-9 (PMC5802549; doi:10.1038/s41408-017-0040-9)
Supplement: Supplementary file 2 — Supplementary table [file 41408_2017_40_MOESM2_ESM.docx]

**Supplementary Table. Multi-amplicon deep sequencing panel (n= 62)**

| Gene name | Gene name | Gene name |
| --- | --- | --- |
| *APC* | *FLT3* | *PRPF8* |
| *ASXL1* | *GATA2* | *PTCH1* |
| *BCOR* | *GLI1/GLI2* | *PTPN11* |
| *BCORL1* | *GNB1* | *RAD21* |
| *BTRC* | *GPR98* | *RNF25* |
| *CALR* | *IDH1* | *RUNX1* |
| *CBL* | *IDH2* | *SETBP1* |
| *CCDC42B* | *IRF4* | *SF3B1* |
| *CDH23* | *JAK2* | *SIMC1* |
| *CEBPA* | *JAK3* | *SMC3* |
| *CFTR* | *KDM6A* | *SRSF2* |
| *CSF1R* | *KIT* | *STAG2* |
| *CUX1* | *KRAS* | *STAT3* |
| *DDX41* | *LUC7L2* | *SUZ12* |
| *DDX54* | *MECOM* | *TET2* |
| *DHX29* | *MED12* | *TP53* |
| *DNMT3A* | *MLL* | *U2AF1* |
| *EED* | *NF1* | *U2AF2* |
| *ERBB4* | *NPM1* | *WT1* |
| *ETV6* | *NRAS* | *ZRSR2* |
| *EZH2* | *PHF6* |  |
| All coding regions were sequenced | |  |
