# Supplementary figures and images for: Mutations in DNMT3A, U2AF1, and EZH2 identify intermediate-risk acute myeloid leukemia patients with poor outcome after CR1
Source: Blood Cancer J. 2018 Jan 10;8(1):4. doi: 10.1038/s41408-017-0040-9 (PMC5802549; doi:10.1038/s41408-017-0040-9)

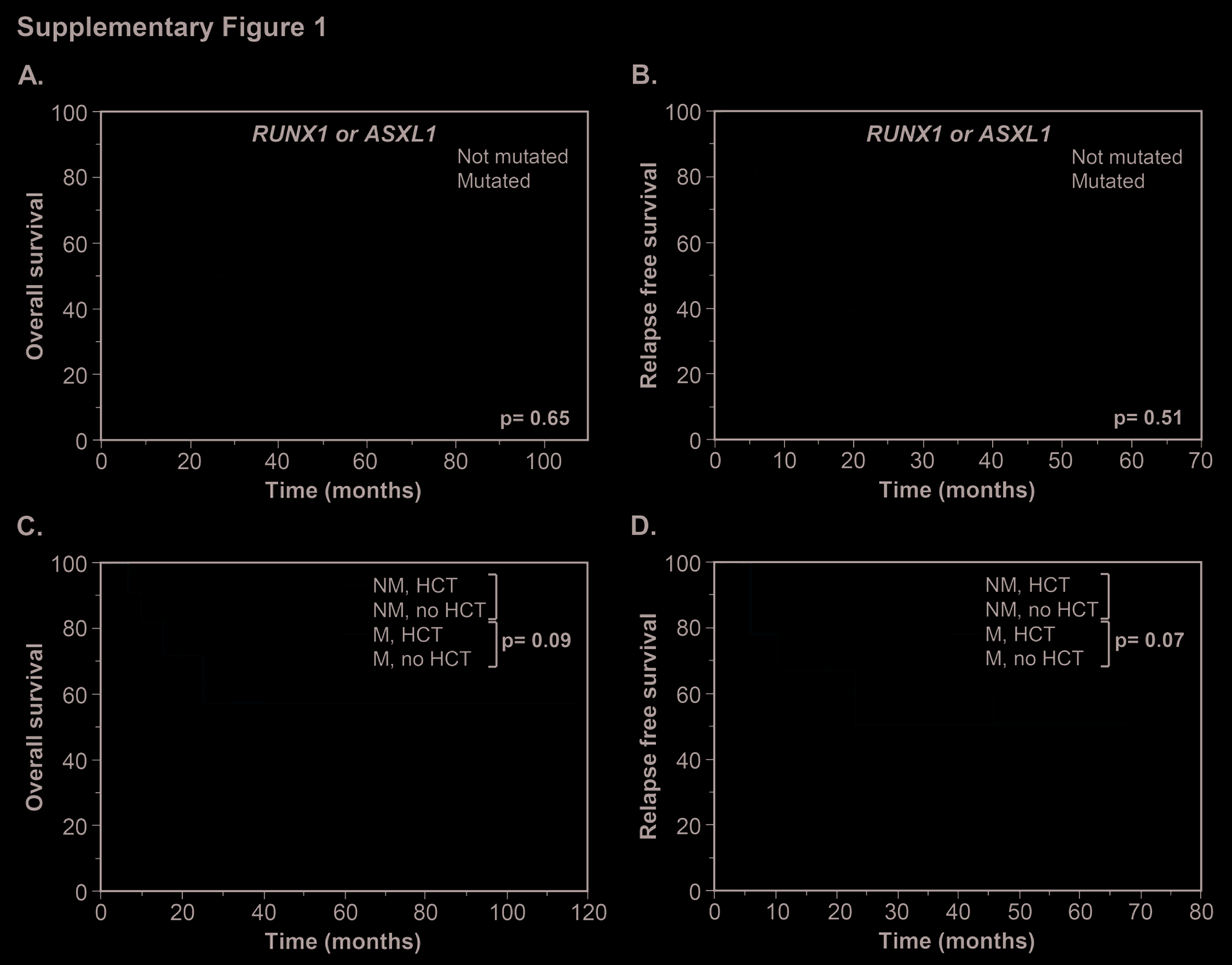

Supplement: Supplementary file 3 — Supplementary Figure 1 [file 41408_2017_40_MOESM3_ESM.tif]

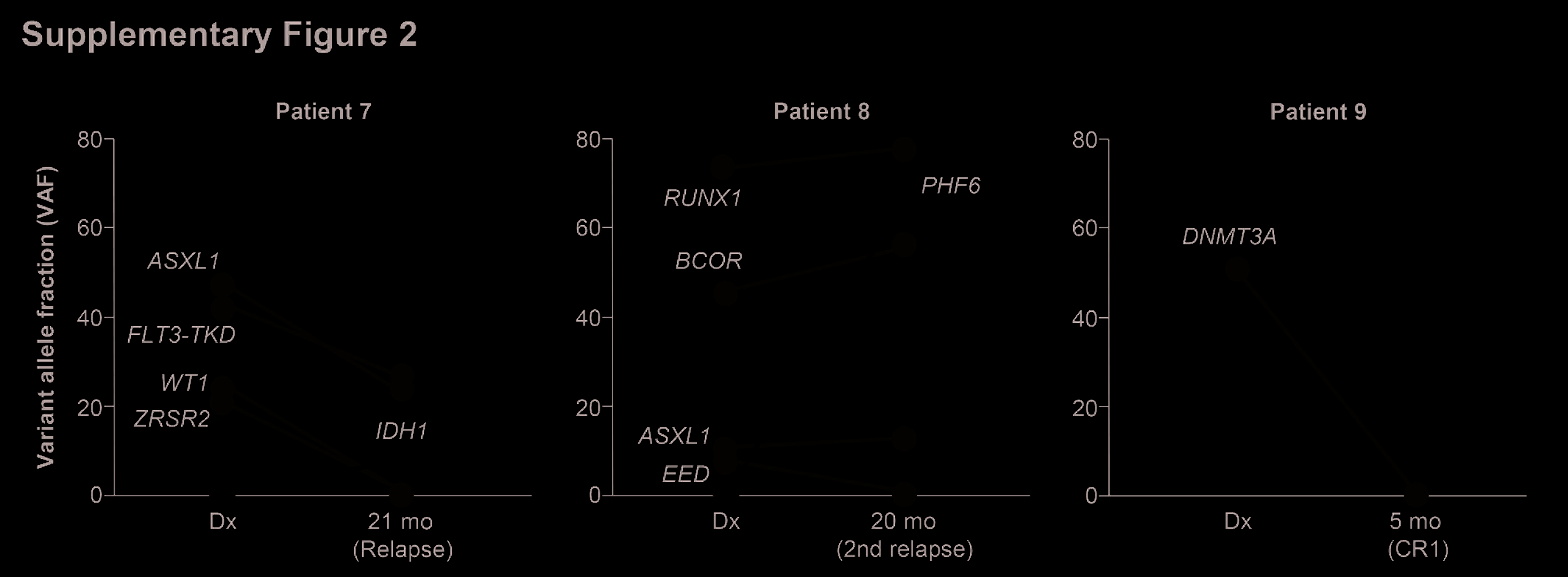

Supplement: Supplementary file 4 — Supplementary Figure 2 [file 41408_2017_40_MOESM4_ESM.tif]
